# Effectiveness of Some Vitamins in the Prevention of Cardiovascular Disease: A Narrative Review

**DOI:** 10.3389/fphys.2021.729255

**Published:** 2021-10-08

**Authors:** Anureet K. Shah, Naranjan S. Dhalla

**Affiliations:** ^1^School of Kinesiology, Nutrition and Food Science, California State University, Los Angeles, Los Angeles, CA, United States; ^2^Department of Physiology and Pathophysiology, St. Boniface Hospital Albrechtsen Research Centre, Max Rady College of Medicine, Institute of Cardiovascular Sciences, University of Manitoba, Winnipeg, MB, Canada

**Keywords:** cardiovascular diseases, vitamin deficiency, vitamin supplements, cardiac dysfunction, cardiac arrhythmias, metabolic abnormalities, oxidative stress

## Abstract

By virtue of their regulatory role in various metabolic and biosynthetic pathways for energy status and cellular integrity, both hydro-soluble and lipo-soluble vitamins are considered to be involved in maintaining cardiovascular function in health and disease. Deficiency of some vitamins such as vitamin A, B_6_, folic acid, C, D, and E has been shown to be associated with cardiovascular abnormalities whereas supplementation with these vitamins has been claimed to reduce cardiovascular risk for hypertension, atherosclerosis, myocardial ischemia, arrhythmias, and heart failure. However, the data from several experimental and clinical studies for the pathogenesis of cardiovascular disease due to vitamin deficiency as well as therapy due to different vitamins are conflicting. In this article, we have attempted to review the existing literature on the role of different vitamins in cardiovascular disease with respect to their deficiency and supplementation in addition to examining some issues regarding their involvement in heart disease. Although both epidemiological and observational studies have shown some merit in the use of different antioxidant vitamins for the treatment of cardiovascular disorders, the results are not conclusive. Furthermore, in view of the complexities in the mechanisms of different cardiovascular disorders, no apparent involvement of any particular vitamin was seen in any specific cardiovascular disease. On the other hand, we have reviewed the evidence that deficiency of vitamin B_6_ promoted KCl-induced Ca^2+^ entry and reduced ATP-induced Ca^2+^-entry in cardiomyocytes in addition to decreasing sarcolemmal (SL) ATP binding. The active metabolite of vitamin B_6_, pyridoxal 5′-phosphate, attenuated arrhythmias due to myocardial infarction (MI) as well as cardiac dysfunction and defects in the sarcoplasmic reticulum (SR) Ca^2+^-transport in the ischemic-reperfused hearts. These observations indicate that both deficiency of some vitamins as well as pretreatments with different vitamins showing antioxidant activity affect cardiac function, metabolism and cation transport, and support the view that antioxidant vitamins or their metabolites may be involved in the prevention rather than the therapy of cardiovascular disease.

## Introduction

**FIGURE 1:**
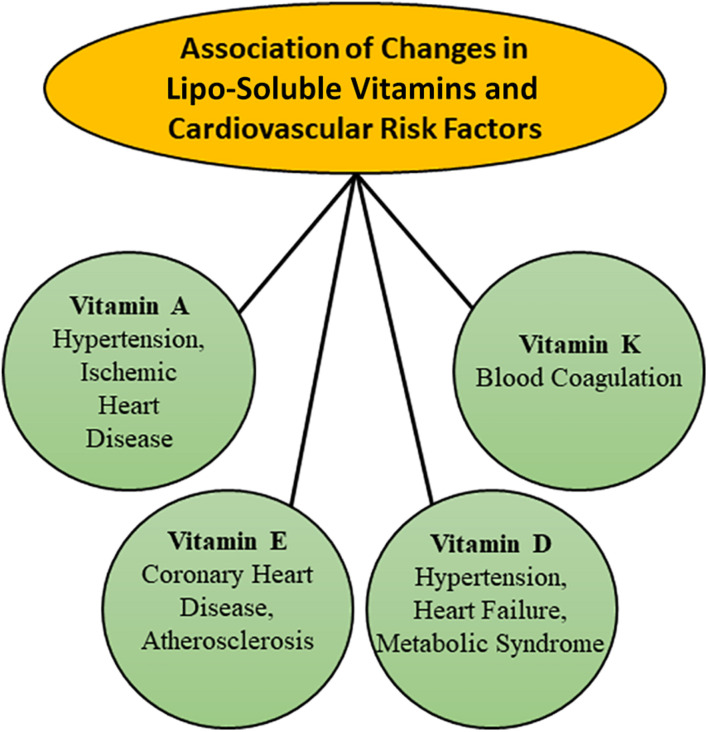
Associations of changes in plasma concentration of different lipo-soluble vitamins with various risk factors for the development of cardiovascular disease.

**FIGURE 2:**
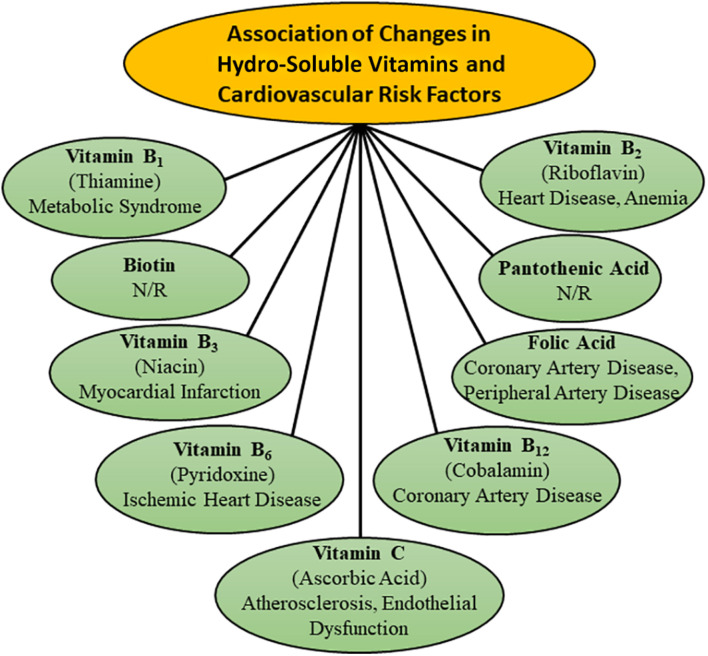
Association of changes in plasma concentration of different hydro-soluble vitamins with various risk factors for the development of cardiovascular disease. N/R- not related.

It is now well known that malnutrition over a prolonged period is one of the major factors, which is associated with the development of heart disease and thus a balanced diet with respect to proteins, carbohydrates and lipids is essential for maintaining cardiovascular health (Ivey, 1979; Farrell, 1980; Alpers et al., 1983; Young and Solomons, 1983; National Research Council (US) Committee on Diet and Health, 1989). It is also known that various proteins, carbohydrates and lipids are metabolized through different but interlinked metabolic pathways in the body for maintaining the integrity and structure of all components of the cardiovascular system as well as providing the required energy for their function. Since both lipo-soluble and hydro-soluble vitamins have been demonstrated to be intimately involved in the regulation of different metabolic processes for cellular biosynthesis and energy production, alterations in their amount are considered to result in cardiovascular abnormalities. It is pointed out that some lipo-soluble vitamins such as A, D, and E, as well as hydro-soluble vitamins such as B_6_ (pyridoxine), B_9_ (folic acid), and C have been reported to play a major role in modulating the cardiovascular function (Chasan-Taber et al., 1996; Palace et al., 1999; Pryor, 2000; Friso et al., 2004; Nemerovski et al., 2009; Moser and Chun, 2016; Stanhewicz and Kenney, 2017; Bilagi, 2018). The implications of changes in both lipo-soluble and hydro-soluble vitamins in different types of cardiovascular diseases such as hypertension, atherosclerosis, ischemic heart disease and heart failure have been depicted in Figures 1, 2, respectively. Although plasma concentrations of most vitamins stay within normal limits in healthy animals and human subjects, deficiencies of both lipo-soluble and hydro-soluble vitamins have been observed in patients with different types of heart disease (Swain and St Clair, 1997; Saremi and Arora, 2010; Pilz et al., 2011; Pawlak, 2015; Eshak and Arafa, 2018; Song and Kang, 2018; Balasubramanian et al., 2019). Thus, various vitamins are commonly recommended for the promotion of cardiovascular health.

Several experimental and observational studies have revealed usefulness of different vitamins in cardiovascular disease (Gale et al., 1995; Wilcken and Wilcken, 1998; Zittermann and Koerfer, 2008; Gori and Münzel, 2011; McGreevy and Williams, 2011; Rafnsson et al., 2011; Czeizel et al., 2013; Dosedel et al., 2021). Links between different vitamins and cardiovascular disease have been established on the basis of their effects on changes in the levels of oxidative stress, inflammation, homocysteine, lipoproteins, and nitric oxide (Rimm et al., 1993; Stanger et al., 2004; Mozos and Marginean, 2015; Farhangi et al., 2017; Stanhewicz and Kenney, 2017; Bartekova et al., 2021). Although both lipo-soluble and hydro-soluble vitamins do not exert direct actions on cardiovascular function, these are considered to affect different cardiovascular diseases upon modulating their respective risk factors. However, their beneficial effects are inconclusive and particularly, the results of various clinical trials for the treatment of heart disease with different vitamins have been disappointing (Olson, 1973; Kushi et al., 1996; Palumbo et al., 2000; Yusuf et al., 2000; Pruthi et al., 2001; Kim et al., 2002; Myung et al., 2013; Ingles et al., 2020). Thus, in view of the uncertainty of demonstrated beneficial cardiovascular effects of different vitamins, the American Heart Association has recommended consumption of vitamin-rich fresh fruits and vegetables instead of vitamins supplements.

It is pointed out that the pathogenesis of different types of cardiovascular diseases is a complex problem and molecular targets of various vitamins may be different for their effectiveness in health and disease. This article is therefore intended to analyze the existing literature to establish if there exists any relationship between a specific vitamin and a specific type of heart disease under conditions of vitamin deficiency or vitamin supplementation. Furthermore, the role of various vitamins will be examined by describing the pathophysiology of heart disease as a consequence of vitamin deficiency. In addition, evidence will be provided to emphasize the potential involvement of various vitamins in the prevention of cardiovascular abnormalities. Since oxidative stress plays a critical role in the development of cardiovascular disease and different vitamins are known to possess antioxidant activity (Dhalla et al., 2000a; Maulik and Kumar, 2012; Neri et al., 2015; van der Pol et al., 2019; Adameova et al., 2020; Bartekova et al., 2021; Ramachandra et al., 2021), the beneficial effects of different vitamins will be evaluated in association with some parameters of oxidative stress.

## Vitamin Deficiency and Cardiovascular Disease

Because of the availability of most vitamins in diet including fruits, vegetables, dairy products and meat preparations, vitamin deficiency is usually not observed in healthy subjects. On the other hand, low levels of some vitamins have been detected in both men and women during the development of different types of cardiovascular abnormalities such as hypertension, atherosclerosis, diabetes, ischemic heart disease, heart failure, and stroke. However, the cause-effects of vitamin deficiency and cardiovascular disease are far from clear. Since lipo-soluble vitamins are stored in different tissues, their levels are not easily depleted whereas hydro-soluble vitamins are readily excreted from the body via the renal system. The following discussion is therefore focused on the deficiencies of only some vitamins and their associations with cardiovascular disease.

### Hydro-Soluble Vitamin Deficiency

Deficiencies of different vitamins such as vitamin B_1_, B_2_, and B_6_ were seen in patients with heart failure in association with depletion of energy stores (Keith et al., 2009). Vitamin B_1_ deficiency has also been associated with cardiovascular disease risk factor such as diabetes, dyslipidemia, obesity, and vascular inflammation (Eshak and Arafa, 2018). Depletion of vitamin B_2_ in cardiovascular disease was associated with anemia and elevated concentrations of homocysteine (Powers, 2003; Balasubramanian et al., 2019). Increased concentrations of homocysteine in patients with coronary artery and peripheral artery diseases were also observed in association with deficiency of vitamins B_6_ and B_12_ (Robinson et al., 1998; Wilcken and Wilcken, 1998). Low levels of vitamin B_12_ and endothelial dysfunction were detected in patients with diabetes, atherosclerosis, myocardial infarction and stroke (Rafnsson et al., 2011; Woo et al., 2014; Pawlak, 2015). Folic acid deficiency has also been shown to occur in vascular disorders as well as congenital heart disease (Swain and St Clair, 1997; Czeizel et al., 2013; Stanhewicz and Kenney, 2017). In addition, deficiency of vitamin B_6_ has been observed in hypertension, atherosclerosis and coronary artery disease (Kok et al., 1989; Chasan-Taber et al., 1996; Herzlich, 1996; Lal et al., 1996). In fact, the animals kept on vitamin B_6_ deficient diet were found to show atherosclerosis (Rinehart and Greenberg, 1949), coronary artery disease (Murray et al., 1978), hypertension (Paulose et al., 1988) and increased sympathetic activity (Viswanathan et al., 1990). Cardiomyocytes obtained from B_6_ deficient rats (Dakshinamurti et al., 1998) showed a marked augmentation of KCl-induced increase in [Ca^2+^]_i_ without any changes in the basal level of [Ca^2+^]_i_ (Table 1). On the other hand, ATP-induced increase in [Ca^2+^]_i_ in cardiomyocytes was depressed due to vitamin B_6_ deficiency and this change was associated with a decrease in sarcolemmal (SL) ATP binding (Table 1). Such alterations in cardiomyocytes from B_6_ deficient animals can be considered to support the view that vitamin B_6_ deficiency may be a risk factor for the development of some cardiovascular disorders.

**TABLE 1:** KCl-induced and ATP-induced changes in [Ca^2+^]_i_ in cardiomyocytes and sarcolemmal (SL) ATP binding in vitamin B_6_ deficient rats.

Parameters	Control	Vitamin B_6_ deficient	Vitamin B_6_ treated
**A. Cardiomyocytes [Ca^2+^]_i_**			
Basal [Ca^2+^]_i_, nM	121 ± 8.6	118 ± 4.4	123 ± 6.4
Increase in [Ca^2+^]_i_ due to 30 mM to KCl, nM	86 ± 5.1	129 ± 8.1*	88 ± 5.5^†^
Increase in [Ca^2+^]_i_ due to 100 μM ATP, nM	69 ± 4.7	33 ± 4.5*	64 ± 4.2^†^
Increase in [Ca^2+^]_i_ due to 10 μM ATP, nM	37 ± 5.8	12 ± 3.6*	–
**B. SL ATP binding**			
ATP binding at 2 nM ATP, pmol/μg protein	3.2 ± 0.25	2.3 ± 0.17*	–
ATP binding at 10 nM ATP, pmol/μg protein	14.0 ± 0.89	9.1 ± 1.2*	–

Control and vitamin B_6_ deficient rats were fed vitamin B_6_-containing or vitamin B_6_-deficient diet for 8 weeks before isolating cardiomyocytes or sarcolemma from the heart. For vitamin B_6_ treated group, 8 weeks vitamin B_6_-deficient rats were injected pyridoxin hydrochloride (10 mg/kg; i.p.) 24 h before the experiment. The data are taken from our paper (Dakshinamurti et al., 1998). *P < 0.05 vs. control; ^†^P < 0.05 vs. vitamin B_6_ deficient group.

Although low levels of plasma vitamin C due to its decreased intake have been reported to be associated with high risk of cardiovascular disease (Ye and Song, 2008; Wang et al., 2013), the relationship between the plasma levels of vitamin C and risk for cardiovascular events is not clear at present. The risk of coronary artery in women was found to increase due to vitamin C deficiency (Osganian et al., 2003) probably as a consequence of increased oxidation of low-density lipoproteins and development of atherosclerosis (Palinski et al., 1989; Yla-Herttuala et al., 1989). On the other hand, vitamin C deficiency in elderly people was related to the risk of death from stroke rather than from the coronary artery disease (Gale et al., 1995). Nonetheless, some investigators in a population study of men observed a significant relationship between vitamin C deficiency and risk of acute myocardial infarction (NyyssOnen et al., 1997). It should be pointed out that high sensitivity of C-reactive proteins and vitamin C deficiency were seen to occur during the development of heart failure in adults (Song and Kang, 2018). Furthermore, by virtue of its antioxidant effect (Knekt et al., 2004; Bartekova et al., 2021) plasma level of vitamin C has been reported to predict the incidence of heart failure (Pfister et al., 2011).

### Lipo-Soluble Vitamin Deficiency

Since vitamin D deficiency is the most common nutritional problem, extensive research efforts have been made to understand its relationship with cardiovascular disorders as well as mechanisms of its impact on cardiovascular function. Several investigators have emphasized that vitamin D deficiency plays a critical role in the pathogenesis of cardiovascular disease including hypertension, heart failure and ischemic heart disease (Wang et al., 2008; Judd and Tangpricha, 2009; Agarwal et al., 2011; Kheiri et al., 2018; Latic and Erben, 2020). It is noteworthy that congestive heart failure in vitamin D deficiency was associated with impaired systolic and diastolic functions, hypertension, and peripheral vascular disease (Kheiri et al., 2018). Low levels of vitamin D were observed to favor the development of atherosclerosis and myocardial infarction as a consequence of inflammation, autoimmunity, endothelial dysfunction, formation of foam cells, and proliferation of smooth muscle cells (Welles et al., 2014; Mozos and Marginean, 2015; Gominak, 2016). Vitamin D deficiency has also been associated with diabetes, obesity, dyslipidemia, metabolic syndrome, and hypertension (Zittermann, 2006; Perez-Castrillon et al., 2007; Muscogiuri et al., 2017). Both diabetes and hypertension as a consequence of vitamin D deficiency were related to the development of insulin resistance, elevated levels of parathyroid hormone, activation of renin-angiotensin system, abnormal nitric oxide regulation as well as increased oxidative stress and inflammatory pathway (Ku et al., 2013; Sziva et al., 2020; de la Guia-Galipienso et al., 2021). The involvement of vitamin D deficiency in cardiovascular disease is supported by experimental studies showing myocardial hypertrophy, arterial hypertension and increased activity of the renin-angiotensin system in vitamin D receptor knockout mice (Pilz et al., 2011).

Unlike deficiencies of other nutrients, vitamin E deficiency in human is rare because of the sufficient consumption of commonly available food (Olson, 1973; Pruthi et al., 2001). However, in infants and people with fat malabsorption or some genetic conditions, vitamin E deficiency has been recognized (Oski and Barness, 1969; Traber, 1999). On the other hand, varying degrees of anemia, myocardial cell damage, and cardiomyopathy due to vitamin E deficiency have been observed in various species of animals (Madsen et al., 1933; Gullickson, 1949; Gatz and Houchin, 1951; MacKenzie and MacKenzie, 1953; Fitch, 1968). Rabbits fed on vitamin E deficient diet showed several electrocardiographic abnormalities; these alterations in the heart were accompanied by a reduction in high energy phosphate and glycogen stores (Mulder et al., 1954). Heart failure associated with marked metabolic changes were also seen in animals maintained on vitamin E deficient diet (Lu et al., 1941; Gullickson and Calverley, 1946; Draper et al., 1952; Fedelesova et al., 1971). In addition, marked alterations in SL Na^+^-K^+^ ATPase and sarcoplasmic reticular (SR) Ca^2+^-pump ATPase as well as SR Ca^2+^-uptake and Ca^2+^-release activities were decreased in vitamin E deficient rat heart (Fedelesova et al., 1971). It should be mentioned that cardiac abnormalities due to vitamin E deficiency were accompanied by muscular dystrophy (Grigoreva and Medovar, 1959; Read and Nehorayan, 1959; Dhalla et al., 1971; Fedelesova et al., 1971). In view of the antioxidant activity of vitamin E (Bartekova et al., 2021), it is likely that the cardiac abnormalities seen in experimental animals due to vitamin E deficiency are due to the development of increased levels of oxidative stress.

## Vitamin Supplementation and Cardiovascular Disease

Excellent reviews for the management of cardiovascular disease by various vitamins are available in the literature (Olson, 1973; Palace et al., 1999; Stanger et al., 2004; Moser and Chun, 2016; Georgiopoulos et al., 2017). However, the results regarding the supplementation of these nutrients in both animals and human subjects with heart disease are not conclusive. Several epidemiological and observational studies as well as animal experimentations support the use of different vitamins in diverse cardiovascular disorders but well controlled clinical studies have failed to observe their beneficial effects in any of the cardiovascular diseases. It should be noted that most of the randomized clinical trials with vitamins have been carried out to determine the therapeutic aspect of their effectiveness in delaying the progression or reducing the extent of cardiovascular diseases but much effort has not been devoted to investigate their actions in depressing the incidence of disease development. Furthermore, most of these investigations may not have employed the optimal dose of a specific vitamin for a specific disease. Thus, it is difficult to interpret such results with respect to the beneficial effects of different vitamins for the prevention or treatment of any cardiovascular disease.

### Hydro-Soluble Vitamin Supplementation

Different B vitamins have been observed to show beneficial effects in preventing various cardiovascular diseases. Higher intake of vitamin B_6_, B_12_, folic acid, and riboflavin were found to decrease the risk of hypertension and lower the blood pressure in patients with hypertension (Liu et al., 2017, 2018; Psara et al., 2020). Vitamins B such as riboflavin, thiamine, folic acid as well as vitamin B_6_ and vitamin B_12_ have also been found useful in clinical trials for the management of heart failure (Witte et al., 2005; Azizi-Namini et al., 2012; van der Pol et al., 2019). Administration of both folic acid and vitamin B_12_ were reported to attenuate the isoproterenol-induced myocardial cell damage, as well as lower the homocysteine and oxidative stress levels in hyperhomocysteinemic rats (Hagar, 2002). Folic acid also reversed the endothelial dysfunction due to depletion of tetrahydrobiopterin in rabbit aortic rings (Moat et al., 2006). In fact, folic acid supplementation has also been shown to improve the endothelial dysfunction in patients with cardiovascular disease (Stanhewicz and Kenney, 2017).

Prevention of cardiovascular diseases by B vitamins may be associated with the treatment of hypertriglyceridemia as niacin was observed to decrease total cholesterol and triglycerides (Feingold, 2000). Vitamin B complex containing B_1_, B_2_, and B_6_ as well as vitamin B_12_ and folic acids were found to reduce atherosclerosis and ischemic heart disease by their anti-inflammatory actions (Hodzic, 2018). Both folic acid and vitamin B_12_ have been reported to delay the early onset of coronary artery disease by reducing plasma homocysteine levels (Pancharuniti et al., 1994). Furthermore, folic acid and antioxidant vitamins reduced the risk of endothelial dysfunction in patients with coronary artery disease (Title et al., 2000; Long et al., 2020). Administration of folic acid as well as vitamin B_6_ and B_12_ reversed the endothelial dysfunction in patient with hyperhomocysteinemia due to methionine loading (Haynes, 2002), In fact, treatment of hyperhomocysteinemia with these vitamins is considered to be the mainstay therapy (Guthikonda and Haynes, 2006). However, it should be pointed out that meta-analysis of data from several clinical trials with vitamin B_6_, vitamin B_12_, and folic acid did not show any evidence of their protective effects for the progression of atherosclerosis (Bleys et al., 2006).

It should be pointed out that pyridoxal 5′-phosphate (PLP), an active metabolite of vitamin B_6_, was shown to possess excellent potentials for the treatment of ischemic heart disease (Serfontein et al., 1985; Ellis and McCully, 1995; Dhalla et al., 2000a; Kandzari et al., 2005; Dhalla et al., 2013). In this regard, it is noteworthy that PLP was found to prevent the formation of oxyradicals and lipid peroxidation due to H_2_O_2_ (Kannan and Jain, 2004). Furthermore, this agent was shown to depress ATP-induced increase in [Ca^2+^]_i_ in cardiomyocytes as well as SL ATP-binding (Wang et al., 1999). Not only did PLP reduced the I/R-induced cardiac dysfunction, it was also observed to reduce infarct size (Dhalla et al., 2000b; Kandzari et al., 2005). Administration of PLP was shown to decrease ischemia injury in patients subsequent to coronary angioplasty and coronary bypass surgery (Kandzari et al., 2003; Tardif et al., 2007). However, PLP did not show beneficial effects in a large clinical trial in high risk patients undergoing coronary artery bypass graft surgery (Carrier et al., 2008; Mehta et al., 2008). While the exact reasons for the failure of PLP in preventing different cardiovascular events in advanced ischemic heart disease are not clear, pretreatment of animals with PLP has been demonstrated (Dhalla et al., 2013) to attenuate arrhythmias, incidence of ventricular tachycardia and mortality (Table 2) due to myocardial infarction (MI). In addition, I/R-induced cardiac dysfunction as well as changes in SR Ca^2+^-uptake and Ca^2+^-release activities (Table 3) were prevented by pretreatment of rats with PLP (Dhalla et al., 2013). Thus, in view of these observations, it is evident that PLP may prove beneficial in the prevention rather that the therapy of ischemic heart disease.

**TABLE 2:** Effects of vitamin B_6_ and its metabolite, pyridoxal 5′-phosphate (PLP) on myocardial infarction (MI) induced arrhythmias and mortality in rats.

Parameters	Untreated MI	Vitamin B_6_ treated MI	PLP treated MI
ST segment, mV	0.18 ± 0.02	0.19 ± 0.01	0.10 ± 0.02*
QTc interval, ms	554 ± 26	566 ± 31	437 ± 22*
Time of onset of arrhythmias, s	45 ± 9.6	47 ± 8.4	125 ± 11.2*
Number of PVC, %	5.0	5.2	1.4*
Incidence of ventricular tachycardia, %	67	61	12*
Mortality within first day of MI, %	35	30	16*

Treatment of rats with or without vitamin B_6_ (50 mg/kg, daily) or PLP (25 mg/kg, daily) was started 2 days before inducing MI by occluding the coronary artery. The electrocardiographic changes in ST segment, QTc interval, and PVC (premature ventricular contraction) and mortality were monitored during the first day of inducing MI. The data are taken from our paper (Dhalla et al., 2013). *P < 0.05 vs. untreated group.

**TABLE 3:** Effects of vitamin B_6_ metabolite, pyridoxal 5′-phosphate (PLP), on cardiac function and sarcoplasmic reticular (SR) Ca^2+^-transport in rat hearts subjected to ischemia-reperfusion (I/R).

Parameters	Control	Untreated I/R	25 μM PLP treated I/R
**A. Cardiac function**			
LVDP, mm Hg	118 ± 5.4	39 ± 2.1*	96 ± 5.2^†^
LVEDP, mm Hg	7.8 ± 0.6	62 ± 3.7*	25 ± 3.1^†^
+dP/dt, mm Hg/s	2680 ± 134	488 ± 16.7*	2278 ± 87^†^
–dP/dt, mm Hg/s	2346 ± 125	396 ± 15.4*	2042 ± 7.4^†^
**B. SR Ca^2+^-transport**			
Ca^2+^ uptake, nmoles Ca^2+^/mg/min	28.6 ± 0.77	7.9 ± 0.46*	20.4 ± 0.65^†^
Ca^2+^ release, nmoles/mg/15 s	7.2 ± 0.24	2.3 ± 0.18*	5.9 ± 0.23^†^

I/R in isolated perfused rat hearts were induced by 30 min of global ischemia followed by 30 min of reperfusion. PLP was present in the reperfusion medium 10 min before the induction of I/R and was also present throughout the reperfusion period. The data are taken from our paper (Dhalla et al., 2013). *P < 0.05 vs. control group. ^†^*P* < 0.05 vs. untreated group.

Epidemiologic studies have shown that vitamin C reduces atherosclerosis by improving endothelial function and lipid profile as well as inhibiting the oxidation of low density lipoproteins in patients with ischemic heart disease (Moser and Chun, 2016). Higher intake of vitamin C was also shown to decrease the risk of ischemic heart disease in an individual with prevalence of heavy smoking (Nam et al., 2003). Vitamin C administration to patients with ischemic heart disease was observed to restore coronary flow and prevent the reinduction of coronary constriction (McNulty et al., 2007). This vitamin also provided protection against I/R- mediated oxidative stress in human subjects (Davis et al., 2016). The beneficial effects of vitamin C were also seen for attenuating the ischemic heart disease in mice by modulating hyperlipidemia and high density lipoprotein remodeling (Contreras-Duarte et al., 2018). In contrast to its positive effects in the area of ischemic heart disease, several studies regarding the effects of vitamin C on other cardiovascular diseases are controversial. Some investigators have observed reduction in blood pressure in patients with hypertension upon vitamin C supplementation (Rodrigo et al., 2008; Das, 2019) whereas others did not find any reduction in the rate of adverse outcomes related to pregnancy-related hypertension (Roberts et al., 2010). Although vitamin C has been shown to decrease vulnerability of the heart to postoperative atrial fibrillation due to oxidative damage (Rodrigo et al., 2010), promotion of atrial fibrillation due to atrial-tachycardia remodeling in dogs was unaffected by this intervention (Shiroshita-Takeshita et al., 2004). Likewise, negative results were obtained regarding the effects of vitamin C on endothelial dysfunction and atherosclerosis associated with oxidative stress (Antoniades et al., 2003; Gori and Münzel, 2011). Therefore, it appears that vitamin C may be beneficial for the treatment of ischemic heart disease as a consequence of its antioxidant activity but its use for the therapy of other cardiovascular diseases cannot be indicated with certainty at the present time.

### Lipo-Soluble Vitamin Supplementation

Vitamin A and its precursors, α-carotene or β-carotene, were claimed to exert beneficial effects in the development of different cardiovascular diseases (Palace et al., 1999). Increases in the level of serum vitamin A by treatment with this nutrient was shown to decrease both systolic and diastolic blood pressures in patients with hypertension (Chen et al., 2002). Prolonged use of vitamin A was also observed to reduce atherosclerosis in both animals and patients as a consequence of its antioxidant and anti-inflammatory actions (Ozkanlar and Akcay, 2012; Ruiz-León et al., 2019). Supplementation of vitamin A was reported to lower the oxidative stress level in diabetic patients with ischemic heart disease (Altoum et al., 2018). Ischemia-reperfusion (I/R) induced infract size was reduced and the post-ischemic cardiac function recovery was improved by β-carotene in Zucker diabetic rats (Csepanyi et al., 2018), Furthermore, treatment with β-carotene protected the myocardium from advance glycation end product-induced SR stress, apoptosis and autophagy (Zhao et al., 2020). However, β-carotene did not show any beneficial effect on metabolic syndrome in high-fat fed rats (Poudyal et al., 2010).

Administration of vitamin D (calcitriol) in patients with hypertension and heart failure has been shown to exert beneficial effects by inhibiting the renin-angiotensin system and parathyroid hormone secretion, as well as acting directly on vitamin D receptors present in vascular smooth muscle cells, endothelial cells and cardiomyocytes (Nemerovski et al., 2009; Lin et al., 2019). Meta-analysis of several observational studies has revealed that there occurs an inverse relationship between the elevated levels of 25-hydroxyvitamin D (a precursor of calcitriol) and reduction of risk of cardiovascular disease such as myocardial infarction, heart failure and aortic stenosis (Zittermann and Koerfer, 2008; Grandi et al., 2010). Treatment of patients with vitamin D was observed to decrease the progression of coronary artery disease and development of acute myocardial infarction by suppressing the intracellular NF-kB pathway (Legarth et al., 2019). Supplementation of 25-hydroxyvitamin D has been reported to attenuate the development of atherosclerosis by lowering the serum levels of total cholesterol, triglycerides and low density lipoproteins, as well as increasing high density lipoproteins, and endothelial nitric oxide production (Surdu et al., 2021). Vitamin D administration has also been observed to lower different markers of oxidative stress and inflammation in high fat-diet induced obese rats (Farhangi et al., 2017). On the other hand, various clinical studies failed to show any beneficial effects of vitamin D treatment in preventing the ischemic heart disease or reducing its mortality (Bostick et al., 1999; Jarrah et al., 2018; Ingles et al., 2020). In addition, some randomized controlled trials in chronic heart failure and other cardiovascular disease have not shown inconclusive and contradictory results with vitamin D treatment (McGreevy and Williams, 2011; Tsugawa, 2015; Brinkley et al., 2017).

Due to its antioxidant and anti-inflammatory properties as well as its ability to improve immune system and low risk of any adverse effects on human health, vitamin E (α-tocopherol) is most widely used nutritional supplement (Pryor, 2000; Khadangi and Azzi, 2019; van der Pol et al., 2019; Wallert et al., 2019). In fact, several observational and experimental studies have supported the use of vitamin E for the treatment of cardiovascular disease (Pryor, 2000). Supplementation of vitamin E has been reported to reduce blood pressure in patients with essential hypertension (Rodrigo et al., 2008) and prevent complications of pregnancy-associated hypertension (Roberts et al., 2010). Some studies have revealed that treatment with vitamin E delayed the progression and attenuated the extent of atherosclerosis as well as endothelial dysfunction (Salonen et al., 2000; Antoniades et al., 2003; Gori and Münzel, 2011). In fact, α-tocopherol was found to prevent ischemia-reperfusion induced cardiac dysfunction and damage as a consequence of reduction in oxidative stress and inflammation (Nagel et al., 1997; Wallert et al., 2019). Vitamin E supplement also reduced the risk of coronary artery disease in men (Rimm et al., 1993) and produced beneficial effects in ischemic heart disease in mice (Contreras-Duarte et al., 2018). The beneficial effect of vitamin E in myocardial infarction was associated with modulation of different mechanisms (Zarkasi et al., 2019; Ziegler et al., 2020). Pretreatments of rats with vitamin E has also been shown to prevent MI-induced changes in cardiac function as well as ventricular arrhythmias (Sethi et al., 2000). Furthermore, catecholamine-induced arrhythmias, myocardial cell damage, lipid peroxidation and subcellular abnormalities were attenuated by pretreatment of animals with vitamin E (Dhalla et al., 1996; Sethi et al., 2009a,b). These observations suggest that vitamin E is beneficial as a cardioprotective intervention against different pathological stimuli.

In spite of strong support for the beneficial effects of vitamin E, several clinical trials have yielded inconclusive and conflicting results. One study has shown that low doses of vitamin E supplementation decreased the risk of angina in patients without previously diagnosed coronary artery disease whereas high doses decreased myocardial infarction and cardiovascular death (Spencer et al., 1999). The beneficial effects of different doses of vitamin E were dependent upon not only the appropriate dose but also on α- or β- tocopherol forms for inhibiting the release of proinflammatory cytokines as well as activities of 5-lipoxygenase, cyclooxygenase, and tyrosine kinase enzymes (Singh et al., 2005). On the other hand, high doses of vitamin E were also observed to increase the risk of coronary artery disease and myocardial infarction (Wang and Xu, 2019). Conflicting results regarding the beneficial effects of vitamin E treatment for atherosclerosis and coronary artery calcification have been reported in the literature (Antoniades et al., 2003; Machado et al., 2019). Although consumption of vitamin E has been demonstrated to lower the risk of coronary heart disease in middle-aged to older men and women, the primary and secondary cardiovascular events were not affected (Saremi and Arora, 2010). Furthermore, coronary heart disease in postmenopausal women was not modified by vitamin E supplementation (Kushi et al., 1996). In fact, prolonged treatment of high risk patients with vitamin E did not show any effect on different cardiovascular events (Yusuf et al., 2000). However, it is interesting to point out that treatment with vitamin E has been shown to exert beneficial effects on heart disease but certain groups of population and subjects maintained on nutritionally adequate diet do not exhibit additional protection with vitamin E supplement (Chow, 1991). In addition, the protective role of vitamin E in coronary heart disease is evident when patients with no established cardiovascular disease were assessed (Pruthi et al., 2001). Thus, in view of the above-mentioned observations it appears that vitamin E supplementation may play a critical role for the prevention rather than the therapy of cardiovascular disease.

## Conclusion

In this article we have reviewed the pros and cons regarding the role of both lipo-soluble and hydro-soluble vitamins in modifying different cardiovascular diseases such as hypertension, atherosclerosis, ischemic heart disease and heart failure. We have examined the issues related to the association of deficiencies of some vitamins with the development of cardiovascular disease as well as the beneficial effects of some vitamin supplementations for the improvement of cardiovascular function in both humans and animals. Although several epidemiological, observational and experimental studies have revealed beneficial effects of some vitamins showing antioxidant, anti-inflammatory, and auto-immune activities in attenuating cardiovascular disorders, results from well controlled clinical investigations are inconsistent, inconclusive and conflicting. In view of such discrepancies in the experimental and clinical observations, no meaningful conclusion can be made for the use of different vitamins in the treatment of cardiovascular disease. It appears that most of the clinical trials with vitamins in cardiovascular disease have been carried out without measuring their plasma levels before initiating the therapy. It is possible that the beneficial effects of these nutrients may only be seen in patients with low levels of plasma vitamins before starting the treatment and this may have been one of the reasons for the failure of relatively large double blind clinical trials with different vitamins. It is also likely that the use of antioxidant vitamins may be beneficial for the prevention of cardiovascular abnormalities due to different pathological stimuli. This view is based on observations that pretreatment of animals with vitamin B_6_ and vitamin E attenuated the I/R-induced injury or coronary occlusion induced alterations in cardiac function, myocardial metabolism, Ca^2+^-handling by cardiomyocytes, and ventricular arrhythmias. Pretreatment of animals with vitamins A, C, B_6_, and E was also observed to depress the catecholamine-induced ventricular arrhythmias. These positive observations provide an appropriate stimulus for carrying out extensive research work for dose-response and cause-effect relationships with various vitamins to establish their specificity in preventing different cardiovascular diseases. It should be recognized that vitamins do not exert any action in healthy individuals and it is the deficiency of some particular vitamin which leads to the development of a particular cardiovascular abnormality. Thus, it would be prudent to examine the effectiveness of vitamins in patients under conditions when their plasma levels are low. Perhaps some new strategies for the treatment of cardiovascular patients be developed to establish the role of some vitamins as a specific adjunct therapy for a specific disease.

## Author Contributions

Both authors have contributed equally in the preparation of this article and approved its submission for publication.

## Conflict of Interest

The authors declare that the research was conducted in the absence of any commercial or financial relationships that could be construed as a potential conflict of interest.

## Publisher’s Note

All claims expressed in this article are solely those of the authors and do not necessarily represent those of their affiliated organizations, or those of the publisher, the editors and the reviewers. Any product that may be evaluated in this article, or claim that may be made by its manufacturer, is not guaranteed or endorsed by the publisher.

## Abbreviations

SL: sarcolemma
SR: sarcoplasmic reticulum
ATP: adenosine triphosphate
[Ca^2+^]_i_: intracellular concentration of free calcium
ATPase: adenosine triphosphatase
PLP: pyridoxal 5 ′ -phosphate
MI: myocardial infarction
PVC: premature ventricular contraction
I/R: ischemia-reperfusion
LVDP: left ventricular developed pressure
LVEDP: left ventricular end diastolic pressure
+dP/dt: rate of change in contraction
–dP/dt: rate of change in relaxation.
